# Genetic, pathogenic, and antigenic characterization of GX-1, a novel infectious bronchitis virus genotype identified in South Korea

**DOI:** 10.1016/j.psj.2025.106217

**Published:** 2025-12-08

**Authors:** Seung-Ji Kim, Ho-Won Kim, Sun-Min Ahn, Seung-Eun Son, Jin-Ha Song, Yong-Baek Kim, Hyuk-Joon Kwon, Kang-Seuk Choi

**Affiliations:** a Laboratory of Avian Diseases, College of Veterinary Medicine, Seoul National University, Seoul 08826, South Korea; b Research Institute for Veterinary Science, College of Veterinary Medicine, Seoul 08826, South Korea; c Laboratory of Poultry Medicine, Department of Farm Animal Medicine, College of Veterinary Medicine, Seoul National University, Seoul 08826, South Korea; d BK21 FOUR Program For Future Veterinary Medicine Leading Education and Research Center, College of Veterinary Medicine, Seoul National University, Seoul 08826, South Korea; e Laboratory of Clinical Pathology, College of Veterinary Medicine, Seoul National University, Seoul 08826, South Korea

**Keywords:** Infectious bronchitis virus (IBV), Novel Genotype, Spike gene divergence, Pathogenicity and tissue tropism, Antigenic variation

## Abstract

Infectious bronchitis virus (IBV), a highly contagious avian coronavirus, remains a major threat to global poultry production due to its rapid genetic evolution and frequent antigenic shifts. During a nationwide surveillance program in South Korea (2024–2025), we identified a previously unrecognized IBV lineage within dead birds in commercial chicken flocks. The Spike 1 (S1) gene of the IBVs belonging to this lineage exhibited less than 75 % nucleotide identity with those of any previously reported IBV genotypes. Phylogenetic analysis demonstrated that this virus forms a distinct monophyletic clade, with no evidence of recombination detected within the S1 gene. On the basis of these genetic and phylogenetic characteristics, we propose that this virus represents the first lineage of a novel tenth IBV genotype, herein designated GX-1. Complete genome analysis further revealed a high degree of similarity to the recent East Asian GI-19 lineage, except within the Spike (S) gene, suggesting recombination on an East Asian GI-19 genomic backbone. Experimental infection of specific-pathogen-free (SPF) chickens with the proposed GX-1 IBV resulted in milder clinical manifestations, lower lesion severity, and lower mortality compared with infection with the GI-19 IBV isolate. Notably, GX-1 did not induce severe nephritis or renal lesions, indicating a loss of nephropathogenicity, a hallmark of GI-19 IBV infections. Although the virus was detected in respiratory, gastrointestinal, and internal organ tissues, its load and persistence were consistently lower than those of GI-19. Antigenic analysis further revealed limited cross-reactivity between GX-1 and GI-19 IBVs, suggesting distinct serotype characteristics. These results indicate that the proposed GX-1 IBV is a genetically distinct genotype with reduced pathogenicity, altered tissue tropism, and distinct antigenic properties. The emergence of this strain highlights the need for ongoing genomic surveillance and genotype-matched vaccines to manage the evolving IBV landscape.

## Introduction

Infectious bronchitis virus (IBV) is a highly contagious avian coronavirus that continues to impose substantial economic burdens on the global poultry industry (Abozeid, 2023). Although IBV is recognized primarily as a respiratory pathogen, certain isolates exhibit markedly broad tissue tropism, extending beyond the respiratory tract to include the kidneys, intestines, or even the reproductive system (Ali et al., 2025). Consequently, IBV isolates have historically been classified on the basis of their predominant target tissues into pneumotropic, nephropathogenic, enterotropic, or reproductive types (Benyeda et al., 2010; Ali et al., 2024). Such diversity in tissue tropism contributes to the wide range of clinical manifestations observed in infected chickens, which may include respiratory distress, nephritis, enteritis, and reduced egg production.

IBV possesses an ∼27 kb positive-sense, single-stranded RNA genome, with the 5′ two-thirds encoding replicase polyproteins (1a and 1ab) and the 3′ one-third encoding four structural proteins—spike (S), envelope (E), membrane (M), and nucleocapsid (N)—as well as several accessory proteins (3a, 3b, 5a, and 5b) (Cavanagh, 2007). The S protein is cleaved into two subunits, S1, which mediates receptor binding and determines host range and antigenicity, and S2, which facilitates membrane fusion. The S gene, particularly the S1 subunit, is subject to intense immune selection pressure, resulting in high genetic variability (Wickramasinghe et al., 2014). Consequently, IBV is classified into distinct genotypes and lineages according to the sequence of the S1 gene (Valastro et al., 2016). This S1-based classification system captures the antigenic evolution and epidemiological spread of the virus, making it the cornerstone of IBV molecular epidemiology.

Like other RNA viruses, IBV evolves rapidly through point mutation and homologous recombination, contributing to its remarkable antigenic diversity and complicating effective disease control (Cavanagh, 2007). Nine major genotypes comprising more than 32 distinct lineages have been classified on the basis of S1 gene phylogeny, reflecting extensive regional diversification (Rafique et al., 2024). Although several genotypes (e.g., GI-1, GI-13, GI-16, GI-19, and GI-23) have achieved global dissemination, many others remain geographically restricted. In Korea, GI-19 (QX lineage) is the predominant genotype and has been responsible for successive epidemic waves driven by recombinant or antigenic drift variants, including the KM91-like, KII, and K40/09-like sublineages (Kim et al., 2025). GI-15 (KI lineage), another genotype of epidemiological significance, was first identified in Korea in 1986 and is primarily associated with respiratory disease (Lee et al., 2010). Although GI-15 remains geographically restricted to Korea, it continues to circulate in the field, and its recombination with GI-19 has been documented (Kim et al., 2023). These inter-lineage recombination events reflect the remarkable evolutionary plasticity of IBV, enabling the emergence of novel variants with altered tissue tropism and pathogenic potential. A notable example is the GI-23 lineage, which initially emerged in a limited geographic region (India and the Middle East) but rapidly disseminated globally, facilitated by its distinct virological properties (Houta et al., 2021; Ikuta et al., 2023). These dynamic patterns of emergence and dissemination highlight the critical need for continuous molecular surveillance to track the evolution and spread of emerging IBV genotypes (Marandino et al., 2019; Chen et al., 2023).

To monitor the potential genetic diversification of IBV, we conducted a nationwide epidemiological investigation in Korea in 2024–2025, analyzing twenty IBV isolates collected from dead birds on commercial poultry farms affected by respiratory and renal disease outbreaks. Although most isolates were classified within the endemic GI-19 lineage, a subset displayed marked sequence divergence in the spike gene, particularly within the S1 subunit, suggesting the emergence of a previously unrecognized genotype. In this study, we further characterized the genetic, phylogenetic, and pathogenic characterization of this novel genotype, provisionally designated “GX-1″, which represents the first lineage of a newly recognized 10th IBV genotype. Comprehensive analyses, including complete genome sequencing, specific pathogen-free (SPF) chicken infection models and serological assays, were conducted to compare GX-1 with the prevailing GI-19 isolate, with a focus on evolutionary divergence, antigenic profiles, and implications for surveillance and vaccine strategies.

## Materials and methods

### Virus isolation

IBV field isolates were obtained from carcasses submitted to the Avian Disease Laboratory, Seoul National University, for diagnostic investigation of suspected IB outbreaks on commercial chicken farms across various regions of South Korea between 2024 and 2025 N

(Supplementary Table 1). Trachea, kidney, and cecal tonsils were aseptically harvested, homogenized, and diluted to 10 % (w/v) in PBS containing gentamicin (500 µg/mL, Cat. No. G0124. 0010; Ducehfa Biochemie, Netherlands). Viral isolation was conducted by inoculating tissue homogenates into the allantoic cavity of 9- to 11-day-old specific pathogen-free (SPF) embryonated chicken eggs (VALO, Germany), followed by incubation at 37 °C for 72 h. Viral RNA was extracted using a Viral Gene-Spin kit (Cat. No. 17154; iNtRON Biotechnology, Korea), and the presence of IBV was confirmed via reverse-transcription PCR (RT‒PCR) targeting the nonstructural protein 3 (NSP3) gene with previously described primers (Hong et al., 2018). IBV-positive allantoic fluid was aliquoted and stored at −70°C until further use.

### IBV genome amplification and sequencing

The full-length S gene was initially amplified from all IBV-positive samples using the SuperScript™ IV One-Step RT‒PCR System (Cat. No. 12594100; Thermo Fisher Scientific, Waltham, MA, USA), following the manufacturer’s protocol. Amplicons were sequenced on the BIT-seq (BIONICS Indexed Tag) platform (Bionics, Korea), generating approximately 50,000 paired-end reads per fragment. Raw sequencing reads were processed using BBDuk (version 38.84; Brian Bushnell) to remove adapter contamination and low-quality bases. Illumina TruSeq, Nextera, and PhiX adapters were trimmed from the right end with a k-mer length of 27, allowing a single mismatch, and bases with a Phred quality score below 6 were removed from both ends. Overlapping adapter sequences between paired reads were clipped with a minimum overlap of 24 bp, and reads shorter than 10 bp after trimming were discarded. The filtered paired reads were merged, and spike gene sequences were de novo assembled using Geneious Prime v2023.2.1 (Biomatters Ltd., New Zealand), yielding high-quality consensus sequences representing each isolate. The assembled consensus sequences were subsequently compared to reference sequences by BLAST analysis to confirm sequence identity and evaluate assembly accuracy. In addition, a subset of representative strains was selected for complete genome amplification and sequencing. Overlapping tiled amplicons spanning the entire genome were generated using a set of primer pairs (Supplementary Table S2), and sequencing and processing were performed using the same platform and pipeline. Complete genome sequences were constructed and deposited in GenBank under accession numbers PV942399 to PV942404. Individual S gene sequences were also deposited in GenBank under accession numbers PV920038 to PV920057.

### Genotyping and phylogenetic analysis

Multiple sequence alignments were performed for the partial and full-length S1 genes and complete genomes. Alignments were conducted using MAFFT v7.490 within Geneious Prime, incorporating prototype strains from each recognized IBV genotype and lineage, along with representative field isolates and reference sequences (Supplementary Tables S3 and S4). Genotype assignment was based on previously established criteria (Valastro et al., 2016). Phylogenetic trees of the full S1 gene and partial S1 regions (S1-HVRI and II) were reconstructed following the approach described previously (Guzmán et al., 2025). For the whole genome dataset, maximum likelihood (ML) phylogenetic tree was generated using IQ-Tree 2.4.0, and statistical support for internal branches was assessed using 1,000 ultrafast bootstrap replicates (Minh et al., 2020). The phylogenetic trees were visualized using the online tool iTOL v7 (https://itol.embl.de/) (Letunic and Bork, 2024).

### Recombination analysis

Potential recombination events in the genomes of the IBV isolates were investigated using the Recombination Detection Program version 4 (RDP4, v4.101) (Martin et al., 2015). Nine detection methods were applied: RDP, GENECONV, BootScan, MaxChi, Chimera, SiScan, PhylPro, LARD, and 3Seq. Recombination signals were considered significant only when supported by at least five methods with p values < 1 × 10⁻¹⁴ (Yan et al., 2021). To visualize potential recombination breakpoints, similarity plots were generated using SimPlot v3.5.1 (Lole et al., 1999).

### Animal experiments

All animal experiments were conducted in filtered isolators located at BioPOA Co. (Hwaseong, Korea). All animal procedures were approved by the Institutional Animal Care and Use Committee (IACUC) of BioPOA Co. under protocol number BP2025-C21-02. The animals were euthanized using CO_2_ inhalation. The IBV isolates used for the challenge experiments included isolate SNU-24007 (Group 1, G1), which represents the novel genotype GX-1, and isolate SNU-24012 (Group 2, G2), which is classified as the endemic GI-19 lineage. 1 Day-old SPF White Leghorn chicks (hatched in-house from SPF embryonated eggs; VALO SPF, Germany) were randomly assigned to three groups: G1 (*n* = 38), G2 (*n* = 38), and the mock-infected negative control group (Group 3, G3; *n* = 25). Birds were housed in filtered isolators located at BioPOA Co. Inoculations were performed via the oculo-nasal route using 0.1 mL of 10⁵ EID₅₀ virus suspension for the infected groups or an equivalent volume of sterile allantoic fluid for the negative control group. Clinical signs and mortality were monitored throughout the study (*n* = 10 in each infected group; *n* = 5 in the control group). Clinical assessments were conducted using a previously described scoring system (Avellaneda et al., 1994), as follows: 0 = no clinical signs; 1 = slight lacrimation, slight shaking, watery feces or tracheal rales; 2 = lacrimation, presence of nasal exudate, depression, water feces, apparent sneezing or cough; 3 = same as 2, but stronger with severe watery feces or swollen head; 4 = death. At 28 days post-infection (dpi), serum samples were collected from the surviving birds that had undergone clinical monitoring throughout the study and were used to evaluate humoral immune responses by serological assays. At 4, 7, 11, and 14 dpi, selected birds (7 per infected group, 5 from controls) were humanely euthanized for tissue and oropharyngeal and cloacal swab collection. The tissues collected for viral load quantification and histopathological examination included the trachea, lung, kidney, ileum, proventriculus, cecal tonsil, and bursa of Fabricius. Viral shedding was assessed using swabs collected at the time of euthanasia and at intermediate time points.

### Viral load quantification by quantitative RT‒PCR

Viral RNA was extracted from tissue homogenates (10 % w/v) and oropharyngeal and cloacal swabs (in 1 mL of PBS) collected from both infected and control (uninfected) chickens using a Viral Gene-spin Kit. Quantitative real-time RT‒PCR (qRT‒PCR) was performed using EzAMP^TM^ HS One-Step RT‒qPCR 2 × Master Mix (Cat. No. EBT-4821, ELPIS-BIOTECH, Korea) with the prime and probe sets which were previously described (Callison et al., 2006). To quantify IBV viral RNA loads, a standard curve was generated using in vitro–transcribed RNA. The RNA synthesis procedure was as follows. First, the IBV target region was amplified using the primer set described by Callison et al. The amplified PCR product was then cloned into a T7 transcription vector using the Cloning Kit for mRNA Template (Cat. No. 6143, Takara Bio, Japan). In vitro transcription and purification were performed using the MEGAscript™ T7 Transcription Kit (Cat No. AM1333, ThermoFisher Scientific, USA), and the synthesized RNA was quantified by spectrophotometry. A tenfold serial dilution of the in vitro–transcribed RNA was used to construct the standard curve (R² > 0.99), which was applied for absolute quantification of viral RNA copy numbers in tissue and swab samples.

### Serological assays

IBV-specific antibody responses were evaluated with indirect ELISA and virus neutralization (VN) assays using the SNU-24007 (proposed GX-1) and SNU-24012 (GI-19) isolates. For ELISA, each purified antigen was coated onto 96-well plates at 1 µg/well and incubated overnight at 4°C. After blocking, serial twofold dilutions of heat-inactivated serum samples were added, followed by the addition of HRP-conjugated anti-chicken IgY and TMB substrate. ELISA titers were determined as the reciprocal of the highest serum dilution producing an OD value of ≥ 0.2 at 450 nm in triplicate wells. Titers were log_2_ transformed prior to statistical analysis.

Virus neutralization was performed in 10-day-old SPF embryonated chicken eggs using the beta method. Serial twofold dilutions of heat-inactivated serum were mixed with 100 EID₅₀ of either the GX-1 or GI-19 isolate and incubated at 37°C for 1 h. The mixtures were then inoculated into the allantoic cavity of SPF embryos. For each group, serum samples were pooled prior to testing, and all assays were conducted in quadruplicate. Embryo viability was monitored daily, and embryos that died within 24 h post-inoculation were excluded from the analysis. At 3 days post-inoculation, the eggs were chilled, and allantoic fluids were aseptically harvested. Viral replication was assessed using qRT‒PCR. Virus neutralization titers were determined as the reciprocal of the highest serum dilution that neutralized 50 % of the virus, as calculated using the Reed and Muench method (Reed and Muench, 1938). Titers were log_2_ transformed prior to statistical analysis. Titers ≤3 were considered negative. The cross-neutralization capacity of the viruses was evaluated using the Archetti and Horsfall formula to determine antigenic relatedness (Archetti and Horsfall Jr, 1950).

### Histopathological and immunohistochemical analyses

Tracheal, lung, and kidney tissues were fixed in 4 % paraformaldehyde, embedded in paraffin, and sectioned at 4 μm. For histopathological analysis, the sections were deparaffinized, rehydrated, and stained with hematoxylin and eosin (H&E) using standard protocols (Fischer et al., 2008). Lesions were evaluated under light microscopy and scored semiquantitatively on the basis of severity and distribution (0 = no lesion; 1 = mild, focal epithelial degeneration or mild inflammation; 2 = moderate, multifocal damage and infiltration; 3 = severe, diffuse necrosis, marked inflammation, and tissue destruction).

For immunohistochemical (IHC) detection of the IBV antigen, 4-μm-thick paraffin sections were deparaffinized, rehydrated, and subjected to antigen retrieval using citrate buffer (pH 6.0) in a microwave. Endogenous peroxidase activity was blocked with 0.3 % hydrogen peroxide in methanol. After blocking with normal horse serum, the sections were incubated overnight at 4°C with a mouse monoclonal anti-IBV antibody (Cat. No. NBP2-31102; Novus Biologicals, CO, USA; IB95, 1:400). Detection was performed using a biotinylated secondary antibody, a VECTASTAIN Elite ABC—HRP kit, and DAB substrate (Cat. No. PK-6100 and SK-4100; Vector Laboratories, CA, USA), followed by hematoxylin counterstaining.

### Statistical analysis

ELISA data were analyzed using one-way analysis of variance (ANOVA) to compare the mean values among the three groups. The homogeneity of variance was assessed using Brown–Forsythe and Bartlett’s tests. When the assumption of equal variance was met, the ANOVA results were interpreted directly. For the virus neutralization titers, which did not meet the assumptions of normality, a nonparametric Kruskal–Wallis test was applied to compare median values among the three groups. For all analyses, statistical significance was set at *P* < 0.05. Data are presented as the mean ± standard deviation (SD) for parametric analyses or as the median (range) for nonparametric analyses. Statistical analyses were performed using GraphPad Prism version 10.3.0 (GraphPad Software, USA).

## Results

### Identification of a novel IBV genotype from Korean poultry

Between 2024 and 2025, a total of 20 IBV field isolates were recovered from carcasses of commercial chickens submitted for disease diagnosis, including layers (*n* = 7), broilers (*n* = 5), broiler breeders (*n* = 3), and others (*n* = 5). The majority of chicken carcasses from which IBV was isolated exhibited coinfections, most frequently with *E. coli,* followed by avian adenoviruses and *Mycoplasma gallinarum*, as well as other avian pathogens (Supplementary Table 1).

**Fig. 1:**
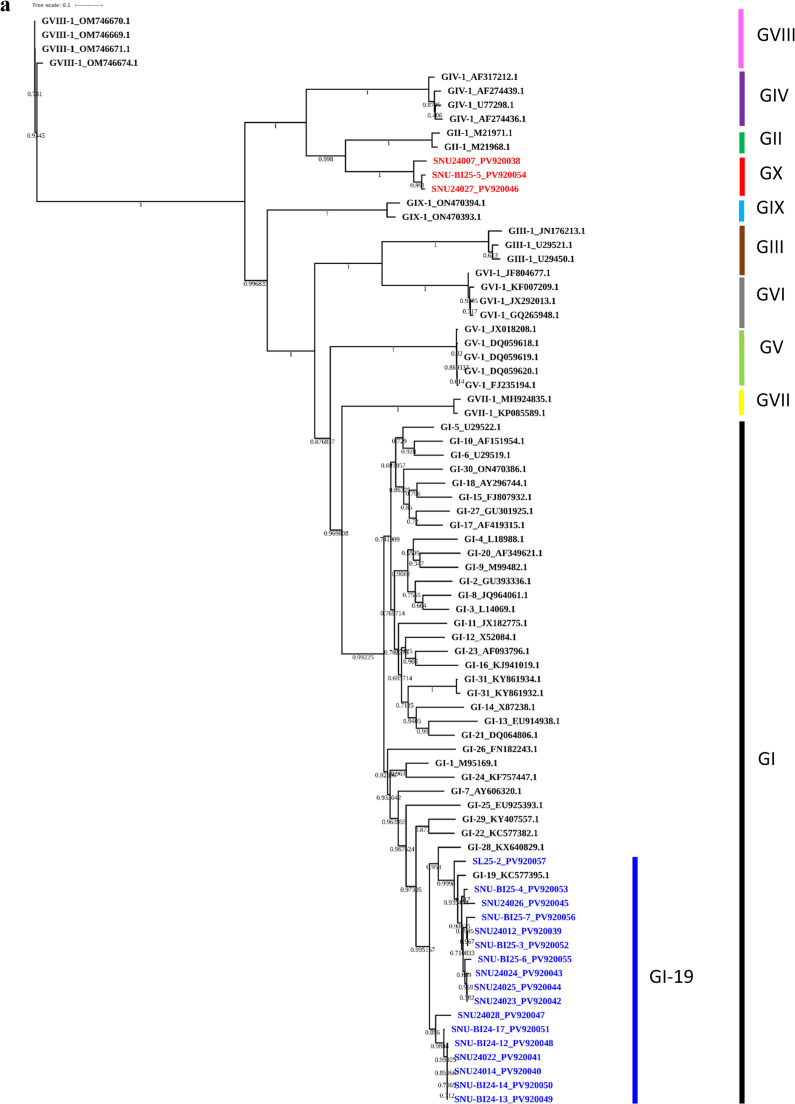

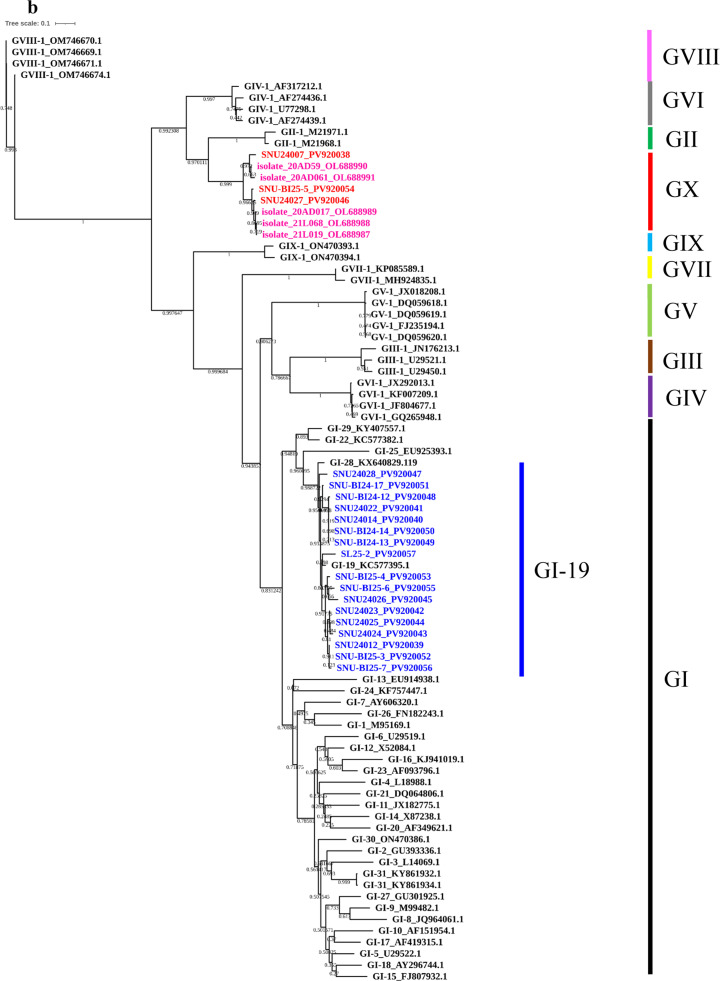

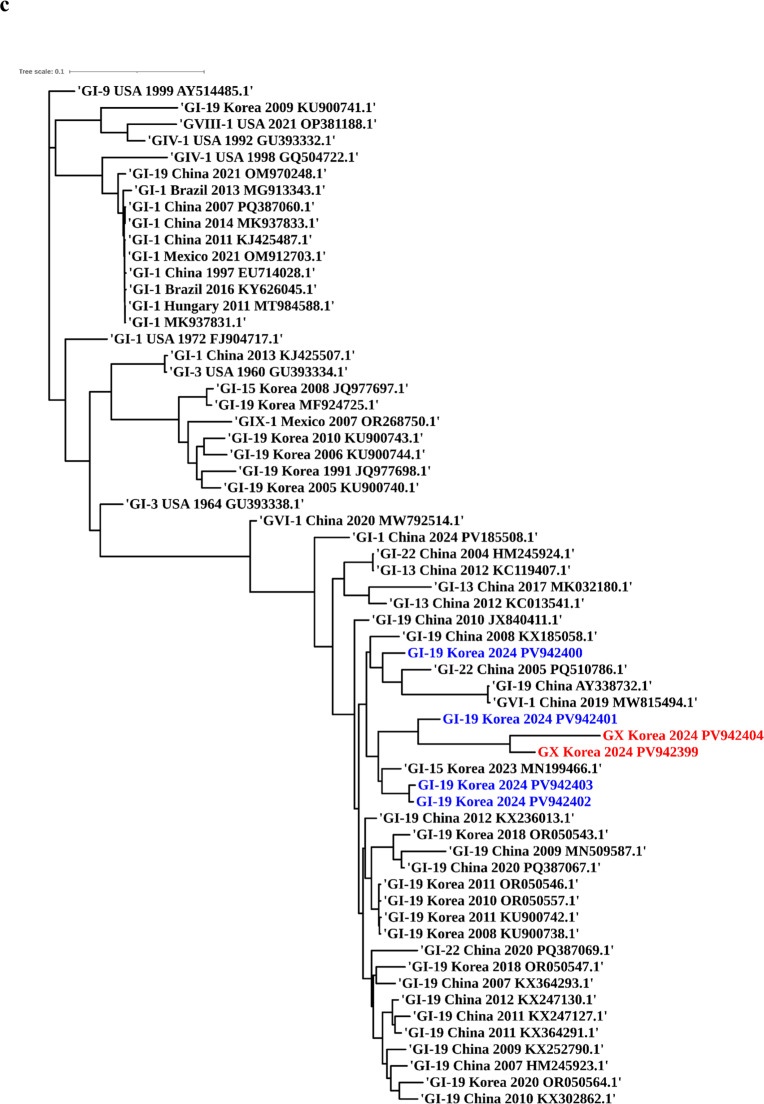
**Maximum-likelihood phylogenetic trees of Korean IBV isolates.** Phylogenetic trees based on (A) the complete S1 gene, (B) the partial S1 gene (nt 1–645, Beaudette strain reference), and (C) the full-genome sequences of the Korean IBV isolates (2024–2025) with reference strains. Korean isolates are color-coded by genotype: GI-19 (blue) and GX-1 (red). Reference strains clustered with GX-1 in the partial S1 tree are highlighted in magenta.

Phylogenetic analyses based on the full-length S1 gene, a partial S1 fragment (nt 1–573, Beaudette numbering), and whole-genome sequences were conducted to assess the genotype, phylogenetic lineage, and potential recombination of IBV isolates in the study. These analyses included representative reference strains encompassing all currently recognized IBV genotypes and lineages. In the S1-based phylogenetic tree, most isolates (*n* = 17) clustered within the GI-19 (QX-like) lineage, confirming its continued predominance in the Korean poultry population. However, three isolates—SNU-24007, SNU-24027, and SNU-BI-25-5—formed a distinct, well-supported monophyletic clade that was phylogenetically distant from all the established genotypes (Fig. 1A). Sequence identity analysis revealed that their S1 genes shared less than 75 % nucleotide identity and less than 70 % amino acid identity with previously characterized genotypes (Table 1), meeting established criteria for novel genotype designation (Valastro et al., 2016). To further explore the genetic relationships of this clade, a separate phylogenetic analysis was performed using the partial S1 region encompassing hypervariable regions (HVRs) I and II (Fig. 1B). This revealed a close clustering of the novel isolates with 20AD17-like strains, which were initially detected in a layer flock in 2020 and subsequently identified in live bird markets offering Korean native chickens in 2021 (Jang et al., 2022). These findings support the grouping of these viruses into a distinct genetic cluster, provisionally designated as genotype GX-1.

**Table 1:** Nucleotide and amino acid identity (%) ranges of the S1 gene among different genotypes.

	Nucleotide identity^1^													
	**SNU24007**	**SNU24027**	**SNUBI25-5**	**GI-1**^3^	**GI-15**^3^	**GI-19**^3^	**GII-1**	**GIII-1**	**GIV-1**	**GV-1**	**GVI-1**	**GVII-1**	**GVIII-1**	**GIX-1**
**SNU24007**		93.6	93.5	59.2	59.5	59.7	71.6	55.8	65	59.8	57.7	58.9	57.6	60.8
**SNU24027**	89.8		98	59.2	59.1	59.1	71.9	56.1	64.7	59.7	56.8	59.7	58.7	60.6
**SNUBI25-5**	90.2	96.6		59.4	59.3	58.7	71.8	55.7	65	59.5	56.4	59.4	58.4	60.2
**GI-1**	51.8	51.6	52.2		77.2	77.5	58.6	62.7	58.9	66.3	63.9	69	57.5	61.4
**GI-15**	51.4	50.2	50.4	73.3		76.4	58	63.1	58.3	66.4	65.5	68.4	57.2	61.6
**GI-19**	52.1	53.1	52.5	76.2	74.3		57.1	63.5	57.4	67.2	65.4	69.5	56.5	60.8
**GII-1**	68.2	69	68.6	51.6	49.7	50.1		55.8	64.7	57.5	56.8	57.5	56.7	60.3
**GIII-1**	45.4	46.6	45.8	56.3	54.5	56.1	47.1		54.3	62.1	68.5	59.9	53.5	56.6
**GIV-1**	60.1	58.5	58.9	49.1	46.4	46.9	59.1	43.7		56	54.9	57.6	57	58
**GV-1**	51.5	51.7	51.7	62.5	62.4	65	49.4	56.3	46.6		63.4	65.2	56.1	60.3
**GVI-1**	47.8	47.8	46.9	58.4	59.6	59.7	49.2	66.4	44.2	57.3		61.8	54.1	58.8
**GVII-1**	49.8	50.5	50.1	66.4	64.7	65.8	50.1	52.6	47.6	61.1	57.2		56.2	63
**GVIII-1**	46.2	47.1	46.9	49	46.5	47.9	45.2	43.2	45.8	46.4	43.9	48.3		57.9
**GIX-1**	51.7	51.9	51.5	54.2	53.9	53.7	50.1	47.9	48.2	53	50.7	58.1	48.2	
	Amino acid identity^2^													

1 Nucleotide identity values between the GX strain and the genotypes are shown in the upper triangle of the matrix.

2 Amino acid identity values between the GX strain and the genotypes are shown in the lower triangle of the matrix.

3 GI-1, GI-15, and GI-19 were selected as representative lineages within Genotype I that have been previously identified in Korea.

**Fig. 2:**
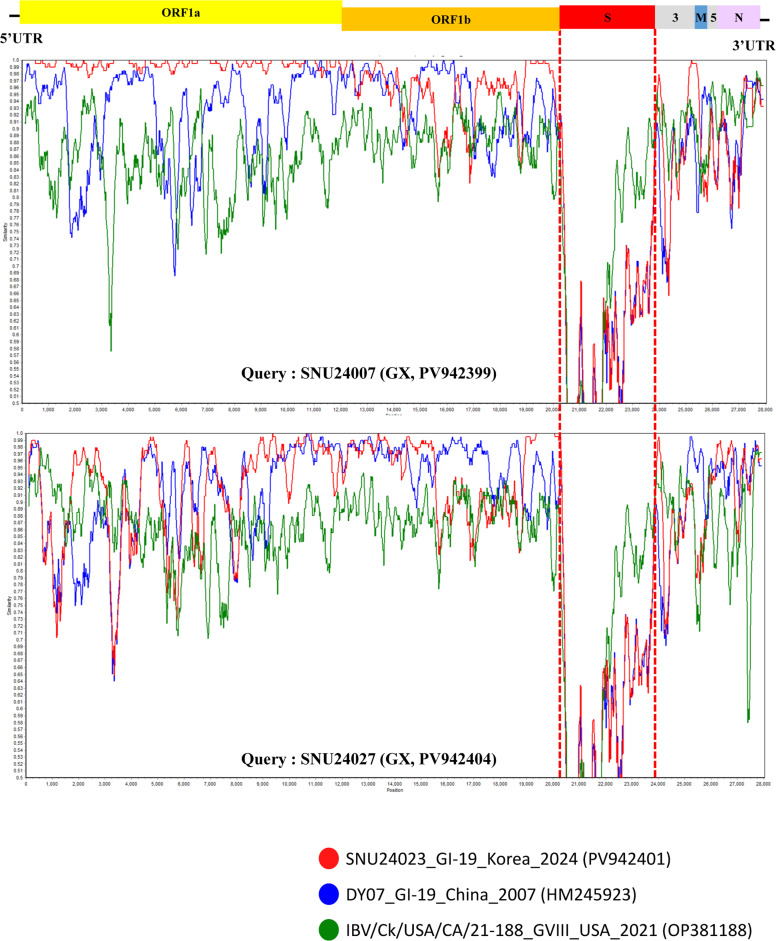
**SimPlot analysis of complete GX strain genome sequences.** Whole-genome SimPlot analysis of the query genome using SNU24023 (red), DY07 (blue), and IBV/Ck/USA/CA/21-188 (green). Among these, SNU24023 and DY07 were identified as potential parental strains by RDP4 analysis. IBV/Ck/USA/CA/21-188, although not considered a parental strain, was included because it exhibited high sequence similarity within the S2 region. The y-axis indicates percentage identity, and the x-axis denotes the nucleotide position across the query genome.

To investigate the evolutionary origin of GX-1, a maximum-likelihood tree based on complete genome sequences was constructed (Fig. 1C). GX-1 isolates grouped within a clade containing GI-19-like isolates from Korea and China, suggesting that GX-1 shares a genomic backbone with GI-19 viruses circulating in East Asia. However, marked divergence was observed in the S gene region, particularly in the S1 subunit, indicating the acquisition of a distinct S gene. To assess whether this divergence arose through recombination, potential recombination events in the full-length S1 gene were analyzed using RDP4 software with nine detection methods (RDP, GENECONV, BootScan, MaxChi, Chimera, SiScan, PhylPro, LARD, and 3Seq). Recombination analysis of the full-length S1 gene using RDP4 revealed no statistically supported recombination signals in this region for GX-1 strains (*p* < 1 × 10⁻¹⁴). In contrast, whole-genome RDP4 analysis identified a consistent recombination pattern involving the spike gene. Although the assignment of major and minor parents differed between the two GX-1–related strains, the inferred parental lineages were consistently associated with the Chinese strain DY07 (2007) and the Korean strain SNU24023 (2024) which were both East-Asian GI-19 lineages. This event corresponded to a complete replacement of the full-length spike gene region, whereas the flanking genomic regions remained most similar to DY07-like sequences. The donor contributing the replaced spike gene could not be unambiguously determined, suggesting that the inserted S gene originated from an uncharacterized lineage (Supplementary data S5). This finding was further supported and visualized by SimPlot analysis, which revealed a sharp decrease in nucleotide identity within the S gene (∼3.5 kb) compared with that of prototype strains, including GI-19, whereas the remainder of the genome retained high similarity to the prototype (Fig. 2).

### Pathogenicity, viral shedding, and immune responses induced by the GX isolate

**Fig. 3:**
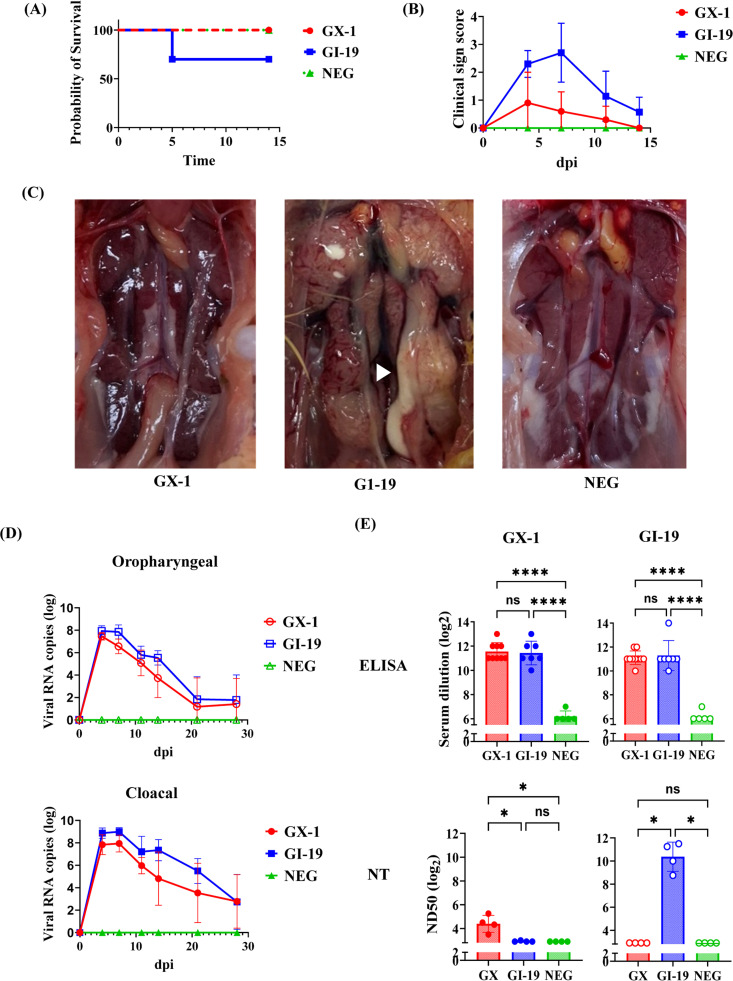
Comparative analysis of survival, clinical signs, kidney gross lesions, viral shedding, and serological responses following infection with bronchitis virus. (A) Survival curves: The GX-1 group (red circles) showed 100 % survival (*n* = 10). The GI-19 group (blue squares) had three mortalities on day 5 post-inoculation, resulting in 70 % survival. The negative control group (green triangles) also showed 100 % survival (*n* = 5). (B) Clinical scores: Mean clinical scores were recorded on days 4, 7, 11, and 14 post-challenge. (C) Gross lesions of kidneys from each group at 7 dpi. The kidney of the GI-19–infected chicken exhibited urate deposition in the ureter, indicated by white arrows. (D) Viral shedding: Viral RNA was detected from oropharyngeal and cloacal swabs. (E) Serology: Antibody responses were measured by ELISA and egg neutralization assays for both homologous and heterologous strains. ELISA results revealed no significant difference between GX-1 and GI-19 (*p* > 0.05), whereas neutralization assays revealed no cross-neutralization between strains. (Statistical significance is indicated as follows: ns = not significant, * = *p* < 0.05, **** = *p* < 0.0001.).

To assess how differences in the spike (S) protein affect pathogenicity and antigenicity within a similar genetic background, the pathogenic potential of the newly identified GX-1 IBV isolate (SNU-24007) which shares a GI-19-like genomic backbone was evaluated in SPF chickens and compared with that of a nephropathogenic GI-19 isolate (SNU-24012). In the GX-1 group, most birds remained asymptomatic or exhibited only mild and transient signs, such as slight feather ruffling and reduced activity. Clinical scores peaked at 0.9 at 4 dpi. In contrast, the GI-19 group showed more severe respiratory symptoms, with clinical scores reaching 2.7 at 7 dpi and three birds dying during the acute phase at 5 dpi (Fig. 3A and B). At necropsy (4 or 7 dpi), GI-19-infected birds displayed severe renal lesions, including renal enlargement, diffuse cortical discoloration, and urate deposition along the ureters. GX-1-infected birds, however, showed no significant renal pathology except mild enlargement and subtle mottling (Fig. 3C). Viral shedding via oropharyngeal and cloacal routes was detected in both groups.

In the GX-1 group, viral loads decreased gradually after 4 dpi but remained detectable until 28 dpi (Fig. 3D). Although viral titers in the GI-19 group were slightly higher, the overall shedding kinetics were similar between the two groups. Humoral immune responses were assessed at 28 dpi (Fig. 3E). In terms of serology, the ELISA titers did not significantly differ between the GX-1 (11.56 ± 0.73) and GI-19 (11.29 ± 1.25) groups. In contrast, VN titers were markedly higher in the GI-19 group (11.50 ± 1.27) than in the GX-1 group (4.40 ± 0.72), indicating a substantially stronger virus-neutralizing response. The result of the two-way cross-neutralization test was presented in supplementary table S6. Cross-neutralization assays revealed that heterologous VN titers were below the detection threshold (<8) in both directions, and calculation of the antigenic relatedness using the Archetti and Horsfall formula yielded an R value of 0.03, confirming the absence of antigenic cross-reactivity between the two genotypes.

### Comparative histopathology and viral antigen distribution between GX and GI-19 infections

**Fig. 4:**
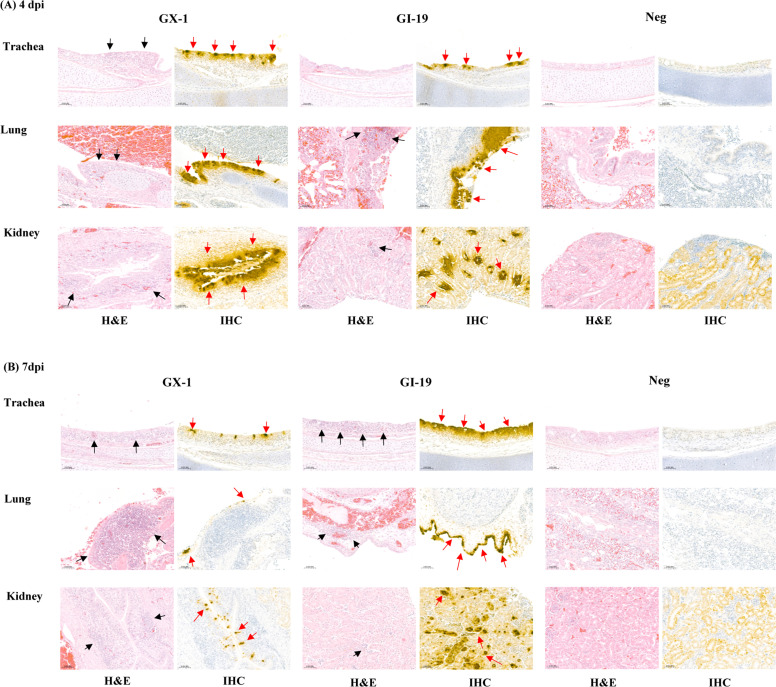
**Histopathologic lesions and immunohistochemical detection of IBV antigens in the trachea, lung, and kidney.** Tissue sections were stained using hematoxylin and eosin (H&E) for histopathology and diaminobenzidine (DAB) for immunohistochemistry (IHC). Chickens were euthanized at 4 days post-infection (dpi) (A) and 7 dpi (B), and representative images of each organ are shown. For direct comparison, H&E and IHC sections from the same organ were prepared in parallel, with a 4 μm interval between sections. In H&E-stained sections, black arrows indicate inflammatory cell infiltration, whereas in IHC-stained sections, red arrows indicate DAB-positive labeling of IBV antigens. Scale bars indicate 50um.

To investigate the tissue-level pathogenesis of the GX-1 and GI-19 isolates, histopathological and immunohistochemical (IHC) analyses were performed on trachea, lung, and kidney tissues collected at 4 and 7 dpi (Fig. 4). Hematoxylin and eosin (H&E) staining revealed epithelial damage and inflammatory cell infiltration in all the organs. In the trachea, both groups exhibited loss of cilia, epithelial desquamation, and submucosal lymphocyte infiltration, with more extensive lesions observed in the GI-19 group. GI-19-infected lungs showed marked interstitial pneumonia with multifocal inflammatory infiltrates, whereas GX-1-infected lungs exhibited only mild bronchial epithelial disruption and limited inflammatory foci. Similarly, kidneys from GI-19-infected birds showed severe tubular epithelial degeneration and necrosis and interstitial nephritis with dense mononuclear infiltration, whereas GX-1-infected kidneys demonstrated only mild tubular epithelial changes with minimal inflammation. IHC analysis revealed positivity for the IBV N protein in the epithelial and stromal cells of the trachea, bronchi, and renal tubules. IHC-positive signals were consistently more intense and widespread in the GI-19 group than in the GX-1 group, corresponding to the observed differences in tissue damage and clinical severity.

### Viral replication kinetics and tissue tropism

**Fig. 5:**
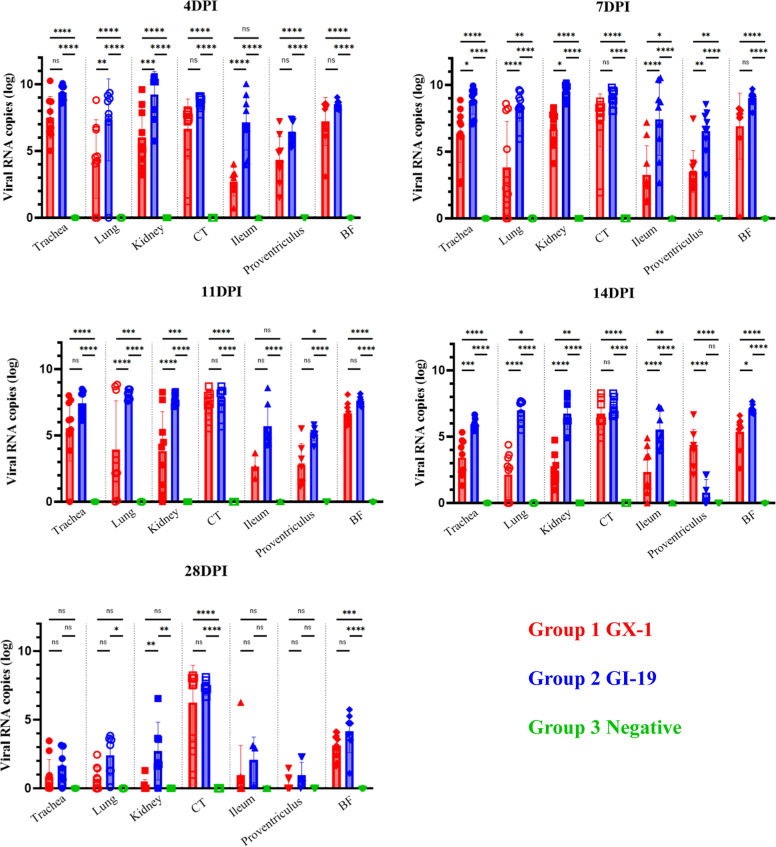
**Viral load comparison across organs in each group at 4, 7, 11, 14, and 28 dpi.** Red, blue, and green lines indicate the GX-1-inoculated group, GI-19-inoculated group, and negative control group, respectively. The y-axis shows viral genome copies (log₁₀ scale), and the x-axis denotes days post-inoculation. (Statistical significance is indicated as follows: ns = not significant, * = *p* < 0.05, ** = *p* < 0.01, *** = *p* < 0.001, **** = *p* < 0.0001.).

Viral RNA loads were quantified by qRT‒PCR at 4, 7, 11, 14, and 28 days post-infection in major organs of infected chickens, including the trachea, lung, kidney, cecal tonsil, ileum, proventriculus, and bursa of Fabricius as shown in Fig. 5. In both groups, viral RNA levels in the trachea, lungs, and kidneys peaked at 4 dpi or 7 dpi but decreased thereafter. Compared with GX-1, the GI-19 isolate exhibited more robust tissue tropism, showing consistently higher viral loads across most organs. In particular, GI-19 displayed significantly higher replication in the kidneys at all examined post-infection time points and in the lungs at all-time points except 28 dpi. By 28 dpi, viral RNA was nearly undetectable in most tissues from both groups, although viral RNA loads in the cecal tonsil were notably higher than those observed in any other examined tissues This persistence was further supported by the detection of viral RNA in cloacal swabs, with oropharyngeal swabs exhibiting substantially lower levels at the same time point.

## Discussion

The identification of the GX-1 genotype highlights that the genomic diversification of IBV in Korea is ongoing despite the longstanding dominance of GI-19 (QX-like) isolates in both field circulation and vaccine formulations (Lee et al., 2021; Hong et al., 2023). Phylogenetic and complete genome analyses revealed that although GX-1 shares a high degree of genomic identity with Korean endemic GI-19 isolates across most of its genome, its S gene is highly divergent. SimPlot analysis confirmed a sharp breakpoint in sequence similarity localized exclusively to the S gene region, supporting the hypothesis of a historical recombination or insertion event with an uncharacterized or genetically distinct donor virus. Importantly, this genetic discontinuity was not accompanied by detectable recombination within the S1 subunit, suggesting that the S gene of the proposed GX-1 IBV may have been acquired during a single ancient recombination event.

The repeated detection of GX-1 isolates in multiple geographically and epidemiologically independent outbreaks across Korea since 2020 supports its classification as a novel IBV genotype (Jang et al., 2022). The absence of closely related intermediate variants implies that the recombination event likely occurred several years ago, followed by cryptic circulation and gradual adaptation within domestic poultry populations. This evolutionary trajectory is reminiscent of that of other coronaviruses, including SARS-CoV-2, where the acquisition of a novel S gene through recombination played a pivotal role in host adaptation and emergence (Graham Rachel and Baric Ralph, 2010; Li et al., 2020).

These alterations in the S gene may contribute to the observed differences in tissue tropism and pathogenicity between the two genotypes. In experimental infection, compared with the virulent GI-19 isolates, GX-1 caused markedly less severe nephritis and other kidney lesions and resulted in lower replication levels in renal tissues, indicating the loss of nephropathogenicity, a hallmark of GI-19 infection (Darwich et al., 2011; Lu et al., 2024). Histopathological analysis of GX-1 infection revealed only mild epithelial damage and reduced inflammatory infiltration in respiratory and renal tissues, further indicating attenuated pathogenicity. Although GX-1 RNA was detected in lymphoid organs such as the cecal tonsils and bursa of Fabricius up to 28 dpi, viral loads were low and decreased gradually, indicating transient persistence without overt pathological consequences. Notably, sequence analysis of the GX-1 isolate (SNU24007) revealed a premature stop codon in the 5b gene, suggesting the presence of a truncated 5b protein. However, previous studies have shown that identical 5b deletions do not necessarily account for reduced virulence; instead, attenuation in IBV has often been linked to alterations in components of the replicase complex (Armesto et al., 2009). Because the replicase gene of GX-1 is highly similar to that of the dominant Korean GI-19 isolates, the attenuated phenotype of GX-1 is unlikely to be explained solely by the 5b truncation. Although the altered tissue tropism and reduced nephropathogenicity of GX-1 may be partly attributed to its unique spike gene characteristics, they could also be influenced by the combined effects of multiple genomic determinants, including genetic variations in regions related to replication efficiency, transcriptional regulation, and viral fitness (Quinteros et al., 2022). In addition, IBV pathogenicity is known to be influenced by the age of the host. Although the present study assessed the virulence of GX-1 isolates using 1-day-old chicks as a standard experimental model, it is noteworthy that these isolates were originally recovered from chickens of various ages. Therefore, further investigations are warranted to evaluate age-dependent differences in pathogenicity across multiple age groups.

Interestingly, viral RNA remained detectable in cloacal swabs beyond the acute phase, suggesting sustained fecal shedding in the absence of clinical disease. This raises important epidemiological concerns; viruses with low pathogenicity but extended shedding capacity may evade surveillance systems that rely heavily on clinical signs for detection (Shaikh et al., 2023). Consequently, GX-1 may serve as a cryptic reservoir within vaccinated populations, facilitating undetected transmission and contributing to prolonged viral circulation. This highlights the importance of surveillance systems capable of detecting not only highly virulent isolates but also low-virulence variants that may persist subclinically within poultry populations, much like low-pathogenicity avian influenza viruses (LPAIVs), which are known to be shed for extended periods without overt clinical signs and often evade symptom-based monitoring (McMenamy et al., 2024).

Experimental infection studies revealed that GX-1 elicits relatively weak immunogenicity in chickens. Notably, cross-neutralization assays showed a lack of reciprocal neutralizing activity between GX-1 and GI-19, indicating substantial antigenic divergence. These results imply that the GX-1 spike protein harbors key antigenic differences that impair recognition by GI-19-induced antibodies, potentially facilitating immune escape (Liu et al., 2007). Given that GI-19-derived vaccines remain the primary immunoprophylactic strategy in Korea, the emergence of an antigenically distinct variant such as GX-1 poses a potential risk to vaccine efficacy (Keep et al., 2020). In theory, GX-1 could exploit this antigenic mismatch to propagate in vaccinated flocks. However, despite its potential for immune escape and its presence in the field since at least 2020, GX-1 has not yet replaced GI-19 as the dominant lineage or triggered large-scale outbreaks. This limited epidemiological footprint suggests that GX-1 may be constrained by other biological factors, such as reduced replication efficiency, suboptimal transmission dynamics, or ecological competition with fitter cocirculating genotypes (Ma et al., 2019). These observations highlight the complex interplay among antigenic drift, viral fitness, and host immunity in shaping IBV population dynamics.

Comparable evolutionary scenarios have been documented for other IBV genotypes, notably GI-23, which expanded beyond its original geographic region following the acquisition of antigenic novelty and partial immune escape (Houta et al., 2021; Ikuta et al., 2023). The identification of GX-1 in multiple Korean provinces, coupled with its genetic distinctiveness and diminished cross-reactivity with GI-19, raises similar concerns about its potential to disseminate more widely if ecological or immunological barriers are lifted. These findings underscore the need to integrate molecular surveillance with routine serological screening and functional assays to ensure timely detection and sound risk assessment. Incorporating GX-1-like isolates into diagnostic algorithms and vaccine seed banks will be pivotal for sustaining IBV control amid an increasingly heterogeneous viral landscape.

In conclusion, the GX-1 genotype represents a genetically distinct and immunologically novel lineage of IBV with attenuated pathogenicity but clear antigenic divergence. Its emergence through recombination, persistence in the field, and ability to partially evade GI-19-based immunity underscore the need to reevaluate current IBV surveillance and vaccine strategies. Continuous monitoring, functional characterization of emerging variants, and the inclusion of antigenically relevant genotypes in future vaccine formulations will be essential to mitigate the threat posed by evolving IBV isolates.

## CRediT authorship contribution statement

**Seung-Ji Kim:** Writing – review & editing, Writing – original draft, Visualization, Software, Project administration, Methodology, Formal analysis, Data curation. **Ho-Won Kim:** Resources, Methodology. **Sun-Min Ahn:** Resources, Methodology. **Seung-Eun Son:** Formal analysis. **Jin-Ha Song:** Formal analysis, Data curation. **Yong-Baek Kim:** Validation. **Hyuk-Joon Kwon:** Validation. **Kang-Seuk Choi:** Writing – review & editing, Supervision, Project administration, Conceptualization.

## Disclosures

The authors declare that there are no conflicts of interest.

## Appendix Supplementary materials

Image, application 1

## References

[bib0001] Abozeid H.H. (2023). Global emergence of infectious bronchitis virus variants: evolution, immunity, and vaccination challenges. Transbound. Emerg. Dis..

[bib0002] Ali A., Farooq M., Altakrouni D., Najimudeen S.M., Hassan M.S.H., Isham I.M., Shalaby A.A., Gallardo R.A., Abdul-Careem M.F. (2024). Comparative pathogenicity of CA1737/04 and mass infectious bronchitis virus genotypes in laying chickens. Front. Vet. Sci..

[bib0003] Ali A., Rahimi R., Mahmoud M.E., Shalaby A.A., Gallardo R.A., Abdul-Careem M.F. (2025). Genetic and phenotypic investigations of viral subpopulations detected in different tissues of laying hens following infectious bronchitis virus infection. Viruses.

[bib0004] Archetti I., Horsfall F.L. (1950). Persistent antigenic variation of influenza A viruses after incomplete neutralization in ovo with heterologous immune serum. J. Exp. Med..

[bib0005] Armesto M., Cavanagh D., Britton P. (2009). The replicase gene of avian coronavirus infectious bronchitis virus is a determinant of pathogenicity. PLoS One.

[bib0006] Avellaneda G.E., Villegas P.., Jackwood M.W., King D.J. (1994). In vivo evaluation of the pathogenicity of field isolates of infectious bronchitis virus. Avian Dis.

[bib0007] Benyeda Z., Szeredi L., Mató T., Süveges T., Balka G., Abonyi-Tóth Z., Rusvai M., Palya V. (2010). Comparative histopathology and immunohistochemistry of QX-like, Massachusetts and 793/B serotypes of infectious bronchitis virus infection in chickens. J. Comp. Pathol..

[bib0008] Cavanagh D. (2007). Coronavirus avian infectious bronchitis virus. Vet. Res..

[bib0009] Callison S.A., Hilt D..A., Boynton T.O., Sample B.F., Robison R., Swayne D.E., Jackwood M.W. (2006). Development and evaluation of a real-time TaqMan RT-PCR assay for the detection of infectious bronchitis virus from infected chickens. J. Virol. Methods..

[bib0010] Chen L., Jiang W., Wu W., Zhang S., Cai J., Lv T., Xiang B., Lin Q., Liao M., Ding C., Ren T. (2023). Insights into the epidemiology, phylodynamics, and evolutionary changes of lineage GI-7 infectious bronchitis virus. Transbound. Emerg. Dis..

[bib0011] Darwich L., Gimeno M., Sibila M., Diaz I., de la Torre E., Dotti S., Kuzemtseva L., Martin M., Pujols J., Mateu E. (2011). Genetic and immunobiological diversities of porcine reproductive and respiratory syndrome genotype I strains. Vet. Microbiol..

[bib0012] Fischer A.H., Jacobson K..A., Rose J., Zeller R. (2008). Hematoxylin and eosin staining of tissue and cell sections. CSH Protoc..

[bib0013] Graham R.L., Baric R.S. (2010). Recombination, reservoirs, and the modular spike: mechanisms of coronavirus cross-species transmission. J. Virol..

[bib0014] Guzmán M., Cádiz L., Sáenz L., Hidalgo H., Verdugo C. (2025). Thirty-five years of IBV evolution in Chile reveals a novel lineage and evidence of vaccine-driven recombination. Viruses.

[bib0015] Hong S.M., An S.H., Lee C.Y., Song C.S., Choi K.S., Kim J.H., Kwon H.J. (2018). Pathobiological and genomic characterization of a cold-adapted infectious bronchitis virus (BP-caKII). Viruses.

[bib0016] Hong S.M., Kim S.J., An S.H., Kim J., Ha E.J., Kim H., Kwon H.J., Choi K.-S. (2023). Receptor binding motif surrounding sites in the spike 1 protein of infectious bronchitis virus have high susceptibility to mutation related to selective pressure. J. Vet. Sci..

[bib0017] Houta M.H., Hassan K..E., El-Sawah A.A., Elkady M.F., Kilany W.H., Ali A., Abdel-Moneim A.S. (2021). The emergence, evolution and spread of infectious bronchitis virus genotype GI-23. Arch. Virol..

[bib0018] Ikuta N., Kipper D., Freitas D.S.S., Fonseca A.S.K., Lunge V.R. (2023). Evolution and epidemic spread of the avian infectious bronchitis virus (IBV) GI-23 in Brazil. Viruses.

[bib0019] Jang I., Thai T.N., Lee J.I., Kwon Y.K., Kim H.R. (2022). Nationwide surveillance for infectious bronchitis virus in South Korea from 2020 to 2021. Avian. Dis..

[bib0020] Keep S., Sives S., Stevenson-Leggett P., Britton P., Vervelde L., Bickerton E. (2020). Limited cross-protection against infectious bronchitis provided by recombinant infectious bronchitis viruses expressing heterologous spike glycoproteins. Vaccines.

[bib0021] Kim H.J., Lee H.C., Cho A.Y., Choi Y.J., Lee H., Lee D.H., Song C.S. (2023). Novel recombinant avian infectious bronchitis viruses from chickens in Korea, 2019–2021. Front. Vet. Sci..

[bib0022] Kim J.Y., Le H.D., Thai T.N., Kim J.K., Song H.S., Her M., Kim H.R. (2025). Revealing a novel GI-19 lineage infectious bronchitis virus sub-genotype with multiple recombinations in South Korea using whole-genome sequencing. Infect. Genet. Evol..

[bib0023] Lee H.C., Jeong S., Cho A.Y., Kim K.J., Kim J.Y., Park D.H., Kim H.J., Kwon J.H., Song C.S. (2021). Genomic analysis of avian infectious bronchitis viruses recently isolated in South Korea reveals multiple introductions of GI-19 lineage (QX Genotype). Viruses.

[bib0024] Lee H.J., Youn H..N., Kwon J.S., Lee Y.J., Kim J.H., Lee J.B., Park S.Y., Choi I.S., Song C.S. (2010). Characterization of a novel live attenuated infectious bronchitis virus vaccine candidate derived from a Korean nephropathogenic strain. Vaccine.

[bib0025] Letunic I., Bork P. (2024). Interactive Tree of Life (iTOL) v6: recent updates to the phylogenetic tree display and annotation tool. Nucleic Acids Res..

[bib0026] Li X., Giorgi E.E., Marichannegowda M.H., Foley B., Xiao C., Kong X.-P., Chen Y., Gnanakaran S., Korber B., Gao F. (2020). Emergence of SARS-CoV-2 through recombination and strong purifying selection. Sci. Adv..

[bib0027] Liu S., Zhang X., Wang Y., Li C., Liu Q., Han Z., Zhang Q., Kong X., Tong G. (2007). Evaluation of the protection conferred by commercial vaccines and attenuated heterologous isolates in China against the CK/CH/LDL/97I strain of infectious bronchitis coronavirus. Vet. J..

[bib0028] Lole K.S., Bollinger R.C., Paranjape R.S., Gadkari D., Kulkarni S.S., Novak N.G., Ingersoll R., Sheppard H.W., Ray S.C. (1999). Full-length human immunodeficiency virus type 1 genomes from subtype C-infected seroconverters in India, with evidence of intersubtype recombination. J. Virol..

[bib0029] Lu Y., Zeng Y., Luo H., Qiao B., Meng Q., Dai Z., Chen N., Zhao L., Meng X., Zhang H., Xia J., Ping J. (2024). Molecular characteristic, evolution, and pathogenicity analysis of avian infectious bronchitis virus isolates associated with QX type in China. Poult. Sci..

[bib0030] Ma T., Xu L., Ren M., Shen J., Han Z., Sun J., Zhao Y., Liu S. (2019). Novel genotype of infectious bronchitis virus isolated in China. Vet. Microbiol..

[bib0031] Marandino A., Vagnozzi A., Craig M.I., Tomás G., Techera C., Panzera Y., Vera F., Pérez R. (2019). Genetic and antigenic heterogeneity of infectious bronchitis virus in South America: implications for control programmes. Avian Pathol.

[bib0032] Martin D.P., Murrell B.., Golden M., Khoosal A., Muhire B. (2015). RDP4: detection and analysis of recombination patterns in virus genomes. Virus Evol..

[bib0033] McMenamy M.J., McKenna R.., Bailie V.B., Cunningham B., Jeffers A., McCullough K., Forsythe C., Cuartero L.G., Flynn O., Byrne C., Connaghan E., Moriarty J., Fanning J., Ronan S., Barrett D., Fusaro A., Monne I., Terregino C., James J., Byrne A.M.P., Lean F.Z.X., Núñez A., Reid S.M., Hansen R., Brown I.H., Banyard A.C., Lemon K. (2024). Evaluating the impact of low-pathogenicity avian influenza H6N1 outbreaks in United Kingdom and Republic of Ireland poultry farms during 2020. Viruses.

[bib0034] Minh B.Q., Schmidt H..A., Chernomor O., Schrempf D., Woodhams M.D., von Haeseler A., Lanfear R. (2020). IQ-TREE 2: new models and efficient methods for phylogenetic inference in the genomic era. Mol. Biol. Evol..

[bib0035] Quinteros J.A., Noormohammadi A..H., Lee S.W., Browning G.F., Díaz-Méndez A. (2022). Genomics and pathogenesis of the avian coronavirus infectious bronchitis virus. Aust. Vet. J..

[bib0036] Rafique S., Jabeen Z., Pervaiz T., Rashid F., Luo S., Xie L., Xie Z. (2024). Avian infectious bronchitis virus (AIBV) review by continent. Front. Cell. Infect. Microbiol..

[bib0037] Reed L.J., Muench H. (1938). A simple method of estimating fifty per cent endpoints. Am. J. Epidemiol..

[bib0038] Shaikh N., Swali P., Houben R.M.G.J. (2023). Asymptomatic but infectious – The silent driver of pathogen transmission. A pragmatic review. Epidemics.

[bib0039] Valastro V., Holmes E.C., Britton P., Fusaro A., Jackwood M.W., Cattoli G., Monne I. (2016). S1 gene-based phylogeny of infectious bronchitis virus: an attempt to harmonize virus classification. Infect. Genet. Evol..

[bib0040] Wickramasinghe I.N.A., van Beurden S.J., Weerts E.A.W.S., Verheije M.H. (2014). The avian coronavirus spike protein. Virus Res..

[bib0041] Yan W., Qiu R., Wang F., Fu X., Li H., Cui P., Zhai Y., Li C., Zhang L., Gu K., Zuo L., Lei C., Wang H., Yang X. (2021). Genetic and pathogenic characterization of a novel recombinant avian infectious bronchitis virus derived from GI-1, GI-13, GI-28, and GI-19 strains in Southwestern China. Poult. Sci..

