# Supplementary material for: Genetic, pathogenic, and antigenic characterization of GX-1, a novel infectious bronchitis virus genotype identified in South Korea
Source: Poult Sci. 2025 Dec 8;105(2):106217. doi: 10.1016/j.psj.2025.106217 (PMC12767831; doi:10.1016/j.psj.2025.106217)
Supplement: Supplementary file 1 [file mmc1.docx]

**Supplementary Table 1. Infectious bronchitis virus isolates obtained from deceased chickens in Korea, 2024–2025.**

| Isolates | Isolated year | Region^1^ | Chicken type^2^ | Age (week) | Gross lesion | Other pathogens  detected ^4^ | Accession no.  (Spike / Full) |
| --- | --- | --- | --- | --- | --- | --- | --- |
| SNU24007 | 2024 | JB | L | 38 | Nasal/Tracheal mucus  Poor follicular development  Renal enlargement | CIAV (CT, Li/SP, Thymus)  MG (Trachea, Turbinate)  FPV (CT, Trachea, scab)  REV (CT, Li/SP, Kidney) | PV920038 / PV942399 |
| SNU24012 | 2024 | CN | B | 4 | Generalized erythema  Nasal hemorrhage  Tracheal hyperemia and mucus | None | PV920039 / PV942400 |
| SNU24014 | 2024 | JN | B | 1 | Trachea mucus  Air sacculitis  Renal urate deposition | None | PV920040 / - |
| SNU24022 | 2024 | GW | BB | 3 | Tracheal hyperemia and mucus  Hepatomegaly and liver necrosis  Pulmonary hemorrhage  Splenic petechiae | *E. Coli* (Liver) | PV920041 / - |
| SNU24023 | 2024 | JN | L | 12 | Tracheal hyperemia and mucus  Air sacculitis  Perihepatitis  Renal enlargement | *E. Coli* (Liver) | PV920042 / PV942401 |
| SNU24024 | 2024 | GG | L | 1 | Thymic atrophy  Liver necrosis | ReoV (Joint, CT) | PV920043 / PV942402 |
| SNU24025 | 2024 | CN | L | 14 | Trachea mucus  Air sacculitis  Hepatomegaly  Renal enlargement | ReoV (Joint, CT)  FAdV (CT) | PV920044 / PV942403 |
| SNU24026 | 2024 | NA | Samgye^3^ | 3 | Nasal discharge  Trachea mucus  Hepatomegaly and liver necrosis  Renal enlargement and urate deposition |  | PV920045 / PV942404 |
| SNU24027 | 2024 | JN | Samgye | 15 | Nasal discharge  Tracheal hyperemia and diphtheritic membrane  Air sacculitis  Poor follicular development  Renal enlargement | FPV (Trachea)  MG (Trachea)  AMPV (Trachea)  FAdV (CT, Li/SP)  *E. Coli* (Trachea) | PV920046 / - |
| SNU24028 | 2024 | GW | L | NA | Trachea mucus  Hepatomegaly and lipidosis  Renal enlagement | *E. Coli* (Liver) | PV920047 / - |
| SNU-BI24-12 | 2024 | NA | B | 4 | Nasal discharge and Trachea mucus  Air sacculitis | MG (Turbinate and Trachea) | PV920048 / - |
| SNU-BI24-13 | 2024 | NA | NA | 3 | Nasal discharge and Facial edema  Trachea mucus  Hepatomegaly and liver necrosis  Renal enlargement | *E. Coli* (Liver) | PV920049 / - |
| SNU-BI24-14 | 2024 | NA | NA | 2 | Femoral head necrosis  Air sacculitis  Hepatomegaly and Perihepatitis  Renal urate deposition | ReoV (Joint, CT)  *E. Coli* (Liver and Joint) | PV920050 / - |
| SNU-BI24-17 | 2024 | GB | B | 5 | Trachea mucus  Hepatomegaly and Hepatic congestion | FAdV (CT, Li/SP) | PV920051 / - |
| SNU-BI25-3 | 2025 | NA | B | 5 | Generalized erythema  Trachea mucus  Air sacculitis  Hepatomegaly and Perihepatitis  Peritonitis | *E. Coli* (Trachea and Liver) | PV920052 / - |
| SNU-BI25-4 | 2025 | GG | L | 69 | Nasal discharge and Trachea mucus  Hepatomegaly and liver necrosis  Focal hepatic necrosis  Splenomegaly and splenic necrosis  Renal enlargement | FAdV (Li/SP and CT)  *E. Coli* (Liver and Spleen) | PV920053 / - |
| SNU-BI25-5 | 2025 | GB | L | 13 | Nasal discharge  Tracheal hyperemia and mucus  Air sacculitis  Hepatomegaly  Renal enlargement | MG (Trachea)  *E. Coli* (Liver) | PV920054 / - |
| SNU-BI25-6 | 2025 | CB | BB | 13 | Trachea mucus  Air sacculitis and Hemoperitoneum  Splenomegaly  Poor follicular development  Renal enlargement | ALV (Li/SP and CT)  REV (Li/SP and CT)  MDV (CT) | PV920055 / - |
| SNU-BI25-7 | 2025 | GB | BB | 22 | Trachea mucus  Hepatomegaly and liver necrosis  Poor follicular development  Renal enlargement and urate deposition | FAdV (CT) | PV920056 / - |
| SL25-2 | 2025 | GG | NKC | NA | Renal enlargement | None | PV920057 / - |

^1^ Regions are abbreviated as follows: JB, Jeollabuk-do; CN, Chungceongnam-do; JN, Jeollanam-do; GW, Gangwon-do; GG, Gyeonggi-do; GB, Gyeongsangbuck-do; NA, Not applicable

^2^ Chicken types are abbreviated as follows: L, Layer; B, Broiler; BB, Broiler Breeder; NKC, Native Korean Chicken

^3^ Samgye : A traditional Korean chicken breed developed through crossbreeding of broiler and layer lines

4 Vira and *Mycoplasma* pathogens detected by PCR are listed along with the organs in which they were identified. Bacterial pathogens are listed only when isolated. Abbreviations of the organas and pathogens are as follows: CT, Cecal Tonsil; Li/SP, Liver and Spleen; CIAV, Chicken Infectious Anemia Virus; MG, *Mycoplasma Gallisepticum*; FPV, Fowl Pox Virus; REV, Reticuloendotheliosis Virus; *E.coli*, *Escherichia coli*; ReoV, Reo Virus; FAdV, Fowl Adenovirus; AMPV, Avian Metapenumovirus; ALV, Avian sarcoma leucosis virus; MDV, Marek disease virus

**Supplementary Table S2. Primers used for infectious bronchitis virus genome sequencing.**

| Fragment | Primer name | 5'-sequence-3' |
| --- | --- | --- |
| FR1 | IBV-full-1F | ACTTAAGTGTGATATAAATATATATCTATTVHAC |
|  | IBV-full-1R | CAAAAGTTTCTTCACCAGATGGT |
| FR2 | IBV-full-2F | GCACTTGTTGATAARAACGGT |
|  | IBV-full-2R | SAAAACCTGCYACTGGCTTAAC |
| FR3 | IBV-full-3F | CTGGAATACGTGTGTTAGAY |
|  | IBV-full-3R | CCAACAAACTCRTTGCCWTC |
| FR4 | IBV-full-4F | GTGTRCATAGTTATAAYAGTG |
|  | IBV-full-4R | GCTTTTCAGAATCTATYTTC |
| FR5 | IBV-full-5F | GAACGTGCTATGACAACWATG |
|  | IBV-full-5R | ACATTAGCAGATGTGGCTTG |
| FR6 | IBV-full-6F | CTTATGGGTTGGGAYTATCC |
|  | IBV-full-6R | CCTTCAACAYTAACACTCATC |
| FR7 | IBV-full-7F | GCTTACAAGAGCTAAGCGTG |
|  | IBV-full-7R | TGCAYTATAAGCAGARGTTTG |
| FR8 (Spike) | IBV-Full-8F | GCACAAGTTTGAYCTTGTG |
|  | IBV-Full-8R | AGGAGTATTGAACCTACGGC |
| FR9 | IBV-full-9F | CATTGCAGTAGGTGTAATTTC |
|  | IBV-full-9R | TTTTTTTGCTCTAACTCTATACTAGCC |

**Supplementary Table S3. Background information on reference strains of infectious bronchitis virus spike genes used in this study.**

| IBV strain | Genotype | Country | Year | Accession No. |
| --- | --- | --- | --- | --- |
| Beaudette | GI-1 | USA | 1937 | M95169.1 |
| Holte | GI-2 | USA | 1954 | GU393336.1 |
| Gray | GI-3 | USA | 1960 | L14069.1 |
| Holte | GI-4 | USA | 1962 | L18988.1 |
| N1/62 | GI-5 | Australia | 1962 | U29522.1 |
| VicS | GI-6 | Australia | 1962 | U29519.1 |
| TP/64 | GI-7 | Taiwan | 1964 | AY606320.1 |
| L165 | GI-8 | USA | 1965 | JQ964061.1 |
| ARK99 | GI-9 | USA | 1973 | M99482.1 |
| B | GI-10 | New Zealand | 1970s | AF151954.1 |
| UFMG/G | GI-11 | Brazil | 1975 | JX182775.1 |
| D3896 | GI-12 | The Netherlands | 1978 | X52084.1 |
| Moroccan-G/83 | GI-13 | Morocco | 1983 | EU914938.1 |
| B1648 | GI-14 | Belgium | 1984 | X87238.1 |
| B4 | GI-15 | Korea | 1986 | FJ807932.1 |
| IZO 28/86 | GI-16 | Italy | 1986 | KJ941019.1 |
| CA/Machado/88 | GI-17 | USA | 1988 | AF419315.1 |
| JP8127 | GI-18 | Japan | 1996 | AY296744.1 |
| 58Hen-93II | GI-19 | China | 1997 | KC577395.1 |
| Qu_mv | GI-20 | Canada | 1996 | AF349621.1 |
| Spain/97/314 | GI-21 | Spain | 1997 | DQ064806.1 |
| 40/GDGZ-97I | GI-22 | China | 1997 | KC577382.1 |
| Variant2 | GI-23 | Israel | 1998 | AF093796.1 |
| V13 | GI-24 | India | 1998 | KF757447.1 |
| CA/1737/04 | GI-25 | USA | 2004 | EU925393.1 |
| NGA/B401/2006 | GI-26 | Nigeria | 2006 | FN182243.1 |
| GA08 | GI-27 | USA | 2008 | GU301925.1 |
| ck/CH/LGX/111119 | GI-28 | China | 2011 | KX640829.1 |
| γCoV/ck/China/I0111/14 | GI-29 | China | 2014 | KY407557.1 |
| Mex-07-1 | GI-30 | Mexico | 2007 | ON470386.1 |
| V819AU_CL_88 | GI-31 | Chille | 1988 | KY861932.1 |
| 19R6AU_CL_86 | GI-31 | Chille | 1986 | KY861934.1 |
| D1466 | GII-1 | The Netherlands | 1979 | M21971.1 |
| N1/88 | GIII-1 | Australia | 1988 | U29450.1 |
| DE/072/92 | GIV-1 | USA | 1992 | U77298.1 |
| N4/02 | GV-1 | Australia | 2002 | DQ059618.1 |
| TC07-2 | GVI-1 | China | 2002 | GQ265948.1 |
| V1397 | GII-1 | The Netherlands | 1980s | M21968.1 |
| N1/08 | GIII-1 | Australia | 2008 | JN176213.1 |
| V6-92 | GIII-1 | Australia | 1992 | DQ490219.1 |
| V18/91 | GIII-1 | Australia | 1991 | U29521.1 |
| GA/5381/99 | GIV-1 | USA | 1999 | AF274439.1 |
| CU82616 | GIV-1 | USA | NA | AF317212.1 |
| AR/6386/97 | GIV-1 | USA | 1997 | AF274436.1 |
| GA/Avial1 | GIV-1 | USA | NA | EU283066.1 |
| GA/13055/00 | GIV-1 | USA | 2000 | AF338719.1 |
| 018 | GV-1 | Australia | 2008 | JX018208.1 |
| NS/03 | GV-1 | Australia | 2003 | DQ059619.1 |
| N1/03 | GV-1 | Australia | 2003 | FJ235194.1 |
| N4/03 (S1 | GV-1 | Australia | 2003 | DQ059620.1 |
| K23/10 | GVI-1 | Korea | 2010 | JF804677.1 |
| SDIB781/2012 | GVI-1 | China | 2012 | KF007209.1 |
| GX-NN09032 | GVI-1 | China | 2009 | JX292013.1 |
| GX-NN130021 | GVII-1 | China | 2013 | KP085589.1 |
| ck/China/I0636/16 | GVII-1 | China | 2016 | MH924835.1 |
| Ck/DE/AC-36213/02/2021 | GVIII-1 | Germany | 2021 | OM746674.1 |
| Ck/DE/AC-14655/01/2016 | GVIII-1 | Germany | 2016 | OM746673.1 |
| Ck/DE/AC-14235/06/2016 | GVIII-1 | Germany | 2016 | OM746672.1 |
| Ck/DE/AC-04987/02/2016 | GVIII-1 | Germany | 2016 | OM746671.1 |
| Ck/DE/AC-04677/03/2016 | GVIII-1 | Germany | 2016 | OM746670.1 |
| Ck/DE/AC-01811/03/2016 | GVIII-1 | Germany | 2016 | OM746669.1 |
| ON470394.1 Mex-56-7 | GIX-1 | Mexico | 2007 | ON470394.1 |
| Mex-14P | GIX-1 | Mexico | 2020 | ON470393.1 |

**Supplementary Table S4. Background information on reference strains of complete genomes of infectious bronchitis virus used in this study.**

| IBV strain | Genotype | Country | Year | Accession No. |
| --- | --- | --- | --- | --- |
| YX10 | GI-19 | China | 2010 | JX840411.1 |
| SNU-8065 | GI-19 | Korea | 2008 | KU900738.1 |
| SNU-11045 | GI-19 | Korea | 2011 | KU900742.1 |
| IBV/Korea/415/2010 | GI-19 | Korea | 2010 | OR050557.1 |
| IBV/Korea/55/2011 | GI-19 | Korea | 2011 | OR050546.1 |
| ck/CH/LSD/120742 | GI-19 | China | 2012 | KX236013.1 |
| ck/CH/LSD/110409 | GI-19 | China | 2011 | KX364291.1 |
| ck/CH/LHB/120402 | GI-19 | China | 2012 | KX247130.1 |
| ck/CH/LHB/111190 | GI-19 | China | 2011 | KX247127.1 |
| DY07 | GI-19 | China | 2007 | HM245923.1 |
| ck/CH/LHLJ/090515 | GI-19 | China | 2009 | KX252790.1 |
| ck/CH/LJL/07III | GI-19 | China | 2007 | KX364293.1 |
| ck/CH/LJS/101111 | GI-19 | China | 2010 | KX302862.1 |
| IBV/Korea/40/2020 | GI-19 | Korea | 2020 | OR050564.1 |
| IBV/Korea/17/2018 | GI-19 | Korea | 2018 | OR050543.1 |
| HF200702-3 | GI-19 | China | 2020 | PQ387067.1 |
| ck/CH/LGS/08I | GI-19 | China | 2008 | KX185058.1 |
| IBV/Korea/61/2018 | GI-19 | Korea | 2018 | OR050547.1 |
| R18/23 | GI-15 | Korea | 2023 | MN199466.1 |
| cK/CH/LDL/091021 | GI-19 | China | 2009 | MN509587.1 |
| HF201226-2 | GI-22 | China | 2020 | PQ387069.1 |
| ZJ051 | GI-22 | China | 2005 | PQ510786.1 |
| Ck/CH/LGD/120723 | GI-13 | China | 2012 | KC013541.1 |
| Ck/CH/LGD/120724 | GI-13 | China | 2012 | KC119407.1 |
| CQ04-1 | GI-22 | China | 2004 | HM245924.1 |
| ck/China/I0737/17 | GI-13 | China | 2017 | MK032180.1 |
| SX2024 | GI-1 | China | 2024 | PV185508.1 |
| I0303/20 | GVI-1 | China | 2020 | MW792514.1 |
| CK/CH/TJ1904 | GVI-1 | China | 2019 | MW815494.1 |
| LX4 | GI-19 | China | NA^1^ | AY338732.1 |
| Arkansas DPI | GI-9 | USA | 1981 | GQ504720.1 |
| Conn46 | GI-1 | USA | 1972 | FJ904717.1 |
| JMK | GI-3 | USA | 1964 | GU393338.1 |
| Gray | GI-3 | USA | 1960 | GU393334.1 |
| ck/CH/LHLJ/131216 | GI-1 | China | 2013 | KJ425507.1 |
| H120 | GI-1 |  | NA | MK937831.1 |
| D1561 | GI-1 | Hungary | 2011 | MT984588.1 |
| ck/CH/LHB/110526 | GI-1 | China | 2011 | KJ425487.1 |
| ZJ971 | GI-1 | China | 1997 | EU714028.1 |
| Ma5 | GI-1 | Brazil | 2016 | KY626045.1 |
| ck/CH/LJL/140820 | GI-1 | China | 2014 | MK937833.1 |
| AH07091 | GI-1 | China | 2007 | PQ387060.1 |
| IBV/ck/MEX/2860/21 | GI-1 | Mexico | 2021 | OM912703.1 |
| AvCoV/Gallus gallus/Brazil/sample 22/2013 | GI-1 | Brazil | 2013 | MG913343.1 |
| CK/CH/GX/202109 | GI-19 | China | 2021 | OM970248.1 |
| Georgia 1998 pass8 | GIV-1 | USA | 1998 | GQ504722.1 |
| Delaware 072 | GIV-1 | USA | 1992 | GU393332.1 |
| IBV/Ck/USA/CA/21-1883 | GVIII-1 | USA | 2021 | OP381188.1 |
| SNU-9106 | GI-19 | Korea | 2009 | KU900741.1 |
| QIA-KR/D79/05 | GI-19 | Korea | 2005 | KU900740.1 |
| QIA-Q43 | GI-19 | Korea | 2006 | KU900744.1 |
| SNU-10043 | GI-19 | Korea | 2010 | KU900743.1 |
| KM91 | GI-19 | Korea | 1991 | JQ977698.1 |
| K2 | GI-19 | Korea | NA | MF924725.1 |
| SNU8067 | GI-15 | Korea | 2008 | JQ977697.1 |
| Mex-56-7 | GIX-1 | Mexico | 2007 | OR268750.1 |

^1^ NA : data not applicable

**Supplementary Data S5. Verification of recombination signals in GX-1 via RDP4 (S1 and whole genome)**


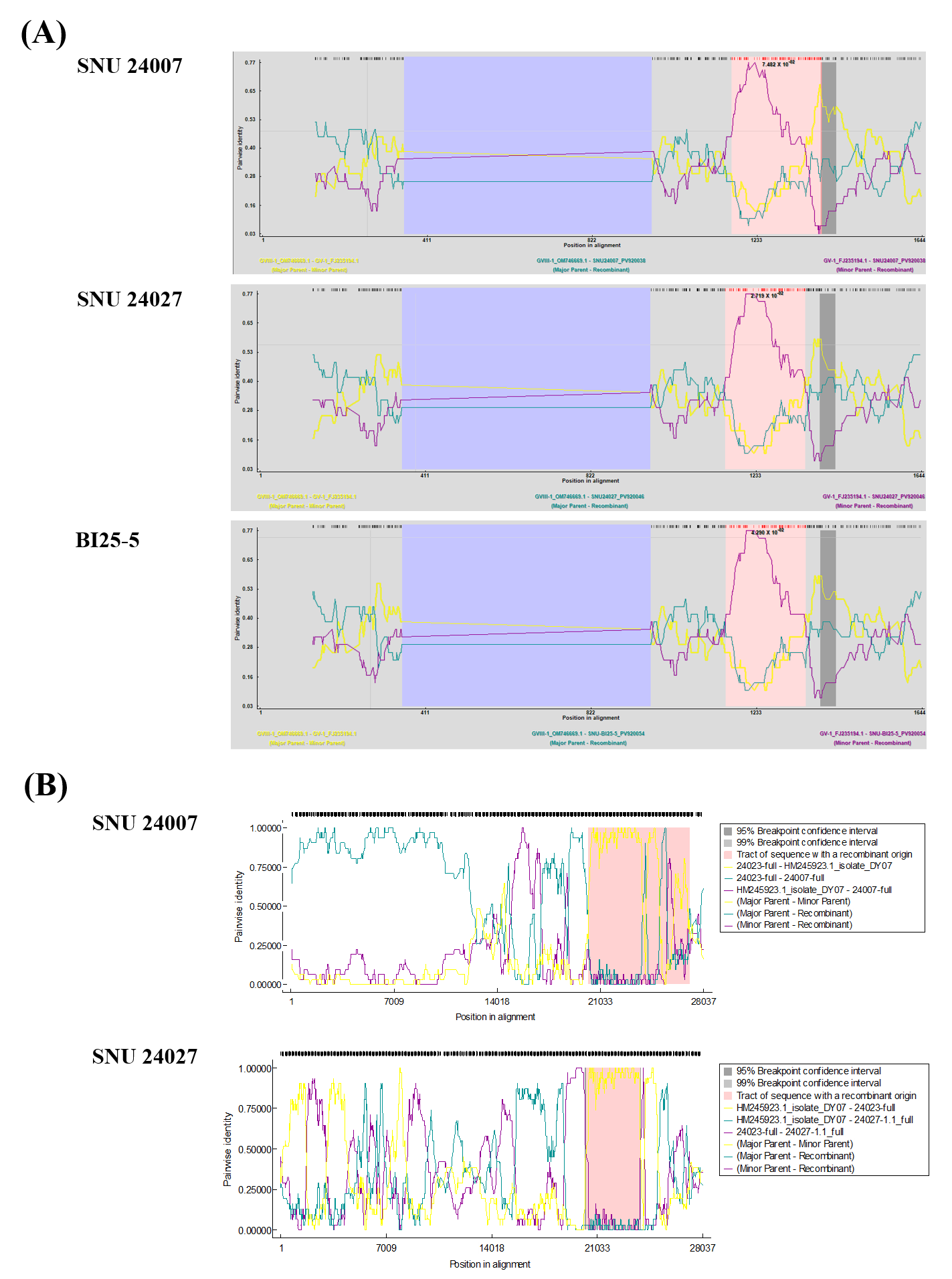


**(C)**

| Methods | Av. P-val | | | | |
| --- | --- | --- | --- | --- | --- |
|  | SNU 24007  Full S1 | SNU 24027  Full S1 | BI25-5  Full S1 | SNU 24007  Whole genome | SNU 24027  Whole genome |
| RDP | 3.415 X 10^-02^ | 3.415 X 10^-02^ | 3.415 X 10^-02^ | **1.326 X 10^-232^** | **1.326 X 10^-232^** |
| GENECONV |  |  |  | **8.512 X 10^-226^** | **8.512 X 10^-226^** |
| BootScan |  |  |  | - | - |
| MaxChi | 4.638 X 10^-02^ | 4.638 X 10^-02^ | 4.638 X 10^-02^ | **6.077 X 10^-44^** | **6.077 X 10^-44^** |
| Chimaera | 3.306 X 10^-03^ | 3.306 X 10^-03^ | 3.306 X 10^-03^ | **2.134 X 10^-41^** | **2.134 X 10^-41^** |
| SiScan | 7.919 X 10^-04^ | 7.919 X 10^-04^ | 7.919 X 10^-04^ | **4.331 X 10^-93^** | **4.331 X 10^-93^** |
| PhylPro |  |  | - | - | - |
| LARD |  |  | - | - | - |
| 3Seq |  |  | - | **3.471 X 10^-193^** | **3.471X10^-193^** |

(A) RDP4-based recombination analysis of the S1 gene in GX-1 strain variants (SNU24007, SNU24027, and BI25-5). Weak breakpoint-like signals were detected, but none reached statistical significance, indicating the absence of recombination breakpoints across the full S1 gene.

(B) RDP4-based recombination analysis of the whole genome in GX-1 strain variants (SNU24007 and SNU24027). A recombination event was identified within the region encompassing the spike protein gene, with SNU24023 and DY07 inferred as the parents.

(C) The table below summarizes the results obtained from the nine recombination detection methods implemented in RDP4. Statistically significant signals, defined as p < 1 × 10⁻¹⁴ in the RDP4 analysis, are highlighted in bold and underlined.

**Supplementary Table S6. Results of two-way cross neutralization test**

| **Neutralization values (log_2_ scale)** | | |
| --- | --- | --- |
| **Antigen**  **Serum** | **GX-1** | **GI-19** |
| **GX-1** | **4. 395±0.7182** | **< 3 ^1^** |
| **GI-19** | **< 3** | **10. 38±1.267** |
| **Negative** | **< 3** | **< 3** |

^1^ The minimum starting dilution was 2³; samples showing no neutralization at 2³ are indicated as <3.
